# Comparative Evaluation of Titanium Feedstock Powder Derived from Recycled Battlefield Scrap vs. Virgin Powder for Cold Spray Processing

**DOI:** 10.3390/ma17051122

**Published:** 2024-02-29

**Authors:** Kiran G. Judd, Kyle Tsaknopoulos, Bryer C. Sousa, Marc Pepi, Danielle L. Cote

**Affiliations:** 1 Department of Mechanical and Materials Engineering, Worcester Polytechnic Institute, Worcester, MA 01609, USA; kgjudd@wpi.edu (K.G.J.); kltsaknopoulos@wpi.edu (K.T.); bcsousa@wpi.edu (B.C.S.); 2 DEVCOM—Army Research Laboratory (ARL), Aberdeen Proving Ground, Aberdeen, MD 21005, USA; marc.s.pepi.civ@army.mil

**Keywords:** gas atomization, titanium, recycled feedstock powder, cold spray, additive manufacturing

## Abstract

Gas-atomization is extensively used to produce metallic feedstock powders for additive manufacturing processes, including gas dynamic cold spray processing. This work explores the potential utility of on-demand recycled titanium scrap feedstock powder as a viable substitute for virgin powder sources. Three recycled titanium powders were atomized from different battlefield scrap sources using a mobile foundry developed by MolyWorks Materials Corporation. Recycled titanium alloy powders were compared against virgin Ti-6Al-4V powder to verify there were no significant variations between the recycled and virgin materials. Powder characterization methods included chemical analysis, particle size distribution analysis, scanning electron microscopy (SEM), Karl Fischer (KF) titration moisture content analysis, X-ray diffraction (XRD) phase analysis, microparticle compression testing (MCT), and nanoindentation. Results indicate that recycled titanium powder provides a viable alternative to virgin titanium alloy powders without compromising mechanical capabilities, microstructural features, or ASTM-specified composition and impurity standards. The results of this work will be used to aid future research efforts that will focus on optimizing cold spray parameters to maximize coating density, mechanical strength, and hardness of recycled titanium feedstock powders. “Cold spray” presents opportunities to enhance the sustainability of titanium component production through the utilization of recycled feedstock powder, mitigating issues of long lead times and high waste associated with the use of conventional virgin feedstock.

## 1. Introduction

Titanium and titanium (Ti) alloys grew to prominence in the late 1950s due to their high performance and unique structure-property relationships. Titanium alloys are advantageous in a versatile range of applications due to their high strength-to-weight ratio, high corrosion resistance, fracture toughness, high-temperature strength, biocompatibility, and processing capabilities [1,2,3,4]. These alloys are classified into five main categories, described as alpha (α), near α, beta (β), near β, and α-β alloys. Alpha and near-α titanium alloys contain low to moderate β-stabilizing elements, retaining the hexagonal close-packed (HCP) α phase as the dominant microconstituent, while metastable and stable β titanium alloys have sufficient β-stabilizer additions to form higher volumes of the body-centered cubic (BCC) β phase upon cooling from the β transus temperature [1,2,4,5]. This variation in alloying content and resultant microstructural constitution leads to differentiated mechanical properties and performances, with α and near-α alloys exhibiting superior fracture toughness and creep strength compared to β and near-β alloys which conversely display higher strength and formability [1,2,4,5]. Two of the most widely used titanium alloys are commercially pure (CP) titanium, an α alloy, and Ti-6Al-4V, an α- β alloy. For reasons previously described, Ti-6Al-4V is one of the most prominently used titanium alloys throughout aerospace, biomedical, automotive, naval, defense, and power generation industries [2,6,7]. 

Conventional production methods for titanium alloys (casting or forging), involving repeated thermo-mechanical processing and extensive machining, result in high costs, long lead times, and significant energy consumption [8]. In contrast, gas atomization is a commonly employed powder production method that involves the disintegration of a molten metal stream into fine droplets using a high-velocity inert gas jet; upon contact with the gas, the metal droplets rapidly solidify into spherical powder particles, resulting in refined microstructures and limited segregation [9]. Controlling gas atomization process parameters allows for tailored particle size distribution, morphology, and purity applicable to a range of additive manufacturing technologies, such as cold spray. Cold spray is a solid-state deposition process that accelerates feedstock powder particles via a heated carrier gas stream through a de Laval nozzle at supersonic velocities before depositing them onto a substrate. This process has been adopted for the remanufacture and repair of coatings and parts across a multitude of industries [10]. Cold spray offers opportunities to overcome the disadvantages of traditional titanium part manufacturing and enables improved sustainability and applicability for titanium alloy production.

The widespread use of titanium alloys in crucial industries, such as aerospace and defense, underlines the need to develop sustainable material sourcing and processing techniques that guarantee continued supply while minimizing environmental ramifications. As the global demand for materials like titanium continues to increase, the sustainability of their production methods becomes a crucial concern. The extraction and processing of virgin titanium are resource-intensive and have significant environmental impacts, including habitat destruction, energy consumption, and greenhouse gas emissions [11]. Recycling titanium scrap and other waste materials helps conserve natural resources by decreasing dependence on mining raw titanium ore. Additionally, recycling aids in securing a consistent titanium supply, which is critical for industries such as defense, aerospace, and biomedical where material availability can be rate-limiting for manufacturing operations. 

Therefore, the present study investigates the viability of using recycled titanium feedstock powder atomized from battlefield scrap for cold spray applications. Three distinct recycled titanium alloy powders were characterized and compared against virgin Ti-6Al-4V powder to verify there were no significant variations between the recycled and virgin materials. Powder characterization methods included chemical analysis, particle size distribution analysis, scanning electron microscopy, Karl Fischer titration analysis, X-ray diffraction phase analysis, microparticle compression testing, and nanoindentation. The objective of this work is to demonstrate the feasibility of generating high-quality, on-demand, recycled titanium feedstock powder and establish a basis for optimizing cold spray deposition parameters using these materials. 

## 2. Experimental Methods and Materials

### 2.1. Feedstock Powders

**Figure 1:**
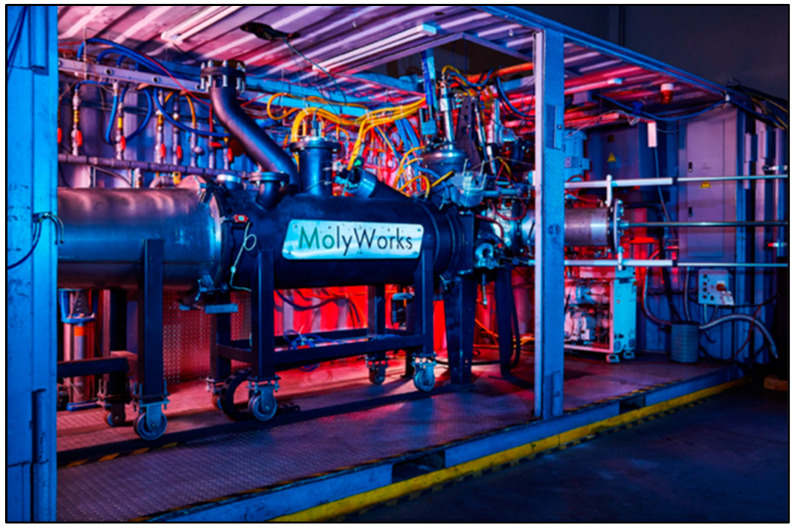
Interior of the Greyhound foundry encapsulated within a shipping container unit for ease of deployability.

Plasma atomized grade 5 virgin Ti-6Al-4V powder was purchased from Advanced Powders and Coating (AP&C) (Boisbriand, QC, Canada). This powder was selected for its ideal characteristics for additive manufacturing applications due to the low levels of entrapped porosity, low satellite content, and notable flowability/packing density. Herein, this powder will be referred to as “virgin Ti-6Al-4V”. Three varying alloys of titanium feedstock powder were produced using gas atomization by the MolyWorks Corporation (Cloverdale, CA, USA) from recycled battlefield titanium armor scrap and commercially pure titanium laser powder bed fusion (LPBF) build plate scrap. The gas atomization process utilized the Molyworks “Greyhound” mobile foundry system that is purposely built to be self-contained in an enclosed 20-foot-long shipping container. Scrap metal is melted in the Greyhound system by a 240-kW plasma torch by which melted material falls through a horizontal argon gas stream to form spheroidized additive manufacturing (AM)-grade powder particles. Figure 1 shows the self-contained atomization unit that is housed inside a shipping container. The recycled titanium atomized feedstocks consisted of a CP Ti, Ti-6Al-4V, and an alloyed blend of the two (~Ti-4.4Al-2.6V). Henceforth, these powders will be referred to as “recycled CP Ti”, “recycled Ti armor alloy”, and “recycled Ti blend alloy”. Titanium armor scrap was sourced from ballistically tested titanium armor plates from the ballistic test range at the DEVCOM—Army Research Laboratory (ARL) (Aberdeen Proving Ground, MD, USA). All ballistic damage and dirt residue were removed from the armor plates prior to atomization. The CP Ti LPBF AM build plates were similarly cleaned and sourced from the DEVOM-ARL Metals AM Laboratory (Aberdeen Proving Ground, MD, USA). 

### 2.2. Powder Characterization

The chemical composition of all titanium powders was measured via inert gas fusion (ASTM E 1409-13), combustion infrared detection (ASTM E 1941-10), and direct current plasma emission spectroscopy (ASTM E 2371-21) (Luvak Laboratories, Inc., Boylston, MA, USA). The oxygen (O), nitrogen (N), and hydrogen (H) contents will be compared between all powders in the Results and Discussion of this work.

Particle size distribution (PSD) and particle morphology were evaluated using a scanning electron microscope (SEM) and particle size/shape analyzer. The particle size distribution was measured in 3-dimensions and assuming spherical particles using a Microtrac TurboSync system (Microtrac Retsch GmbH, Haan/Duesseldorf, Germany). This system quantifies particle size and shape with a hybrid laser diffraction and dynamic image analysis method by an ensemble of particles in an air stream. Additionally, a Zeiss EVO MA-10 SEM (Carl Zeiss AG, Oberkochen, Germany) was used to qualitatively evaluate the size, morphology, and microstructure in 2-dimensions of all titanium powder samples. Loose powder particles were examined by mounting them to SEM stubs with carbon tape and were imaged at 10 kV at varied magnifications ranging from 100× to 2000× with secondary electron emission. The microstructures of all powders were investigated using mechanical and ion beam polishing techniques. A 6-step polishing procedure was used until a mirror finish was attained with a Buehler EcoMet 300 automatic grinder-polisher (Lake Bluff, IL, USA) and an ion-mill polish with argon gas in a JEOL IB-19530CP Cross Section Polisher (Tokyo, Japan) at 8 kV for 2 h to prepare powders for imaging, respectively. Both mechanical and ion beam polishing methods were utilized to ensure all potential microstructural features were identified and whether the observed features were inherent to the material or if artifacts were introduced during sample preparation. The cross-sectional microstructure of each powder was then imaged at 10 kV at varied magnifications with backscattered electron imaging.

The moisture content of all the titanium powders was measured using a coulometric Karl Fischer titration analysis method with a Mettler Toledo Coulometric Karl Fischer C30S Titrator with InMotion KF Oven Autosampler Pro (Columbus, OH, USA). This method places three separate sealed glass vials per titanium powder type, each containing 1–2 g of powder, onto the autosampler. These glass vials were then loaded into the system’s oven where a needle would then pierce the plastic lid to flood the interior of the vial with desiccated air. Afterwards, the vial and encapsulated powder were heated to 220 °C, by which the temperature is sufficient to desorb the moisture from the titanium powder’s surface for 5 min isothermally. The dried air, containing the desorbed moisture, was then carried out of the vial through tubing into the titrator cell. Once the moisture entered the titrator cell, air was bubbled and stirred into a Honeywell high-temperature rated methanol-based analyte “HYDRANAL-Coulomat AG Oven” (Charlotte, NC, USA). An electrode is used to complete the titration process between desorbed moisture introduced into the cell and iodine generated in the solution. The electrode is then used to measure the voltage change of the reaction, thereby converting the voltage measurement into a quantity of moisture introduced into the system (in ppm). This process was repeated for each titanium powder explored in this study, precursed by initial “blank” samples that were empty vials run at identical temperature and time conditions to account for environmental drift effects on the moisture contents. Any drift effects were then subtracted from the final sample moisture contents prior to averaging the results. 

X-ray diffraction (XRD) was executed on all powder samples using a Malvern Panalytical Ltd. Empyrean X-ray Diffractometer (Almelo, Overijssel, The Netherlands), a Cu-Kα radiation source, and Ni filter. Additionally, the data analysis was completed using the HighScore Plus software (Version 4.9.0.27512).

Microparticulate compression testing (MCT) was performed to better understand the particle-scale mechanical property differences across differences in titanium powder compositions and material sourcing, i.e., virgin or recycled scrap feedstocks. Titanium particles were spread across the surface of a diamond compression plate, fixtured to a sliding stage enclosed within a Shimadzu MCT-510 Series system (Kyoto, Japan). Compression strengths were obtained by bringing individual powder particles into focus with two varied magnification objective lenses, in which the particle diameter was measured, and the stage height was focused to the top of the particle for compression. Afterwards, a 50 μm diamond flat-punch indenter probe applied a load at 20.74 mN/s until the target 1600 mN was obtained. As described in [12,13,14], the ultimate tensile strength ($\sigma_{u}$) (MPa) of microparticles was determined from load–displacement data at a displacement equal to 50% particulate compression ($\sigma_{c}(50\%)$). Equation (1) details this calculation:

(1) $$\sigma_{u}=1.4\;(\frac{aP}{\pi d^{2}})$$

whereby, *a* is 2.48 [15], *P* is the test force (N) at a displacement of 50% of the particulate diameter, and *d* is the particle diameter (mm). An average of at least 15 measured powder particles compression strength values was taken for each titanium powder at 50% compression ($\sigma_{c}(50\%)$). 

Nanoindentation testing was performed using an iMicro Pro from the KLA Corp. (Milpitas, CA, USA) on metallographically prepared epoxy-mounted powder particles. An InForce 1000 mN actuator was employed with a diamond Berkovich tip from Micro Star Technologies Inc. (Huntsville, TX, USA) and all indents were applied at the maximum specified load for 15 s. The nanoindenter system and data processing were operated using the InView Software program (Version 19.2.24). Twenty-five particles were tested for each titanium powder type; however, insufficient indents had to be removed during data analysis to ensure only valid indents were being measured. All hardness measurements were reported at depths averaging between 195 nm and 205 nm using a dynamic method, assuring that on average, all indents were compared at a relative depth of 200 nm. A Poisson’s ratio of 0.34 was assumed for all titanium samples tested. Prior to testing the powders, the contact area function and the frame stiffness were determined by testing a fused silica reference standard to obtain the load-depth data. An operational check was also applied to the entire nanoindenter system to check that the actuator and stage mechanics were working properly. Final hardness measurements reported account for any thermal drift, pile-up, and creep-related phenomena that might have occurred during testing. Further information on nanoidentation of single powder particles can be explored here [16,17,18]. 

## 3. Results and Discussion

### 3.1. Chemical Analysis

Table 1 presents the chemical composition of the four titanium feedstock powders examined, as well as the ASTM F1580-18 standard specification for chemical compositions of CP Ti and Ti-6Al-4V powders [19]. Table 1 also compares the measured O, N, C, and H contents among all powders to ensure that both virgin and recycled powders conform to the maximum allowable impurity contents of the ASTM standards. 

Recycled CP Ti, recycled Ti armor alloy, and virgin Ti-6Al-4V powders were all within the allowable composition and impurity specifications for their respective alloy compositions. Alternatively, the recycled Ti blend alloy did not adhere to the ASTM standard for Ti-6Al-4V powder composition, having a lower Al and V content. However, this can be explained by the fact that the blend alloy was a combination of Ti-6Al-4V and CP Ti material. The lack of additional alloying elements in CP Ti resulted in a decreased content of aluminum and vanadium when combined with the Ti-6Al-4V during atomization. Alloying elements aside, the Ti blend alloy was otherwise within the specification when comparing the measured content of O, N, C, and H to those maximum allowable contents for Ti-6Al-4V and CP Ti ASTM standards. 

It was originally hypothesized that the recycled scrap powders would have increased O, N, C, and H contents when compared to their virgin powder counterpart due to the presence of dirt, grease, and environmental containments which could reduce their overall quality and mechanical properties. Nonetheless, the results of the chemical analysis showed that there was no significant difference in elemental composition between recycled and virgin (or ASTM standards) titanium powders. In some cases, the recycled powder, such as the Ti amor alloy, had a lower measured N and C content than the virgin Ti-6Al-4V powder. These results show titanium scrap material can successfully be atomized into usable recycled feedstock powders that are compositionally comparable to virgin powders when using the MolyWorks Greyhound foundry system. 

### 3.2. Particle Size Distribution (PSD) Analysis 

**Figure 2:**
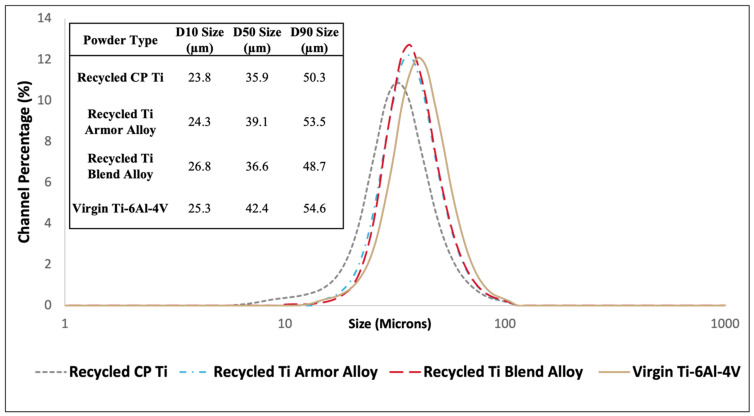
Cumulative particle size distribution and average percentiles of recycled and virgin titanium powders.

Particle size distribution and D10, D50, and D90 percentiles for all powders are shown in Figure 2. The D10, D50, and D90 percentile values refer to the particle diameter sizes (μm) below which 10%, 50%, and 90% of all particles are found, respectively. The channel percentage, located on the y-axis of Figure 2, refers to the probability density function of the data. This reports the percentage of powder particles that are present at a specific size and shows the breakdown of size percentiles. All powders demonstrated similar size distributions which is apparent by the overlapping distribution curves among recycled and virgin powders. Virgin Ti-6Al-4V powder was purchased within the 15–53 μm size range for its optimal size performance for the cold spray process. The PSD measurements confirmed that the virgin Ti-6Al-4V powder was within this desired range and matched the D10, D50, and D90 percentiles reported by the manufacturer. Recycled titanium powders were atomized to be within a similar desired size range as the virgin powder but were on average slightly smaller in size. Quantifiable differences in particle size can be compared by examining the D50 size values among powder types. Broadly, the recycled CP Ti powder was the smallest in size followed by the recycled Ti blend alloy, recycled Ti armor alloy, and the virgin Ti-6Al-4V, respectively. The minor differences between the average particle sizes are thought to have a negligible effect on the resultant deposition efficiency and consolidation quality when cold spraying the virgin and recycled powders. 

### 3.3. Karl Fischer Titration Moisture Content

**Figure 3:**
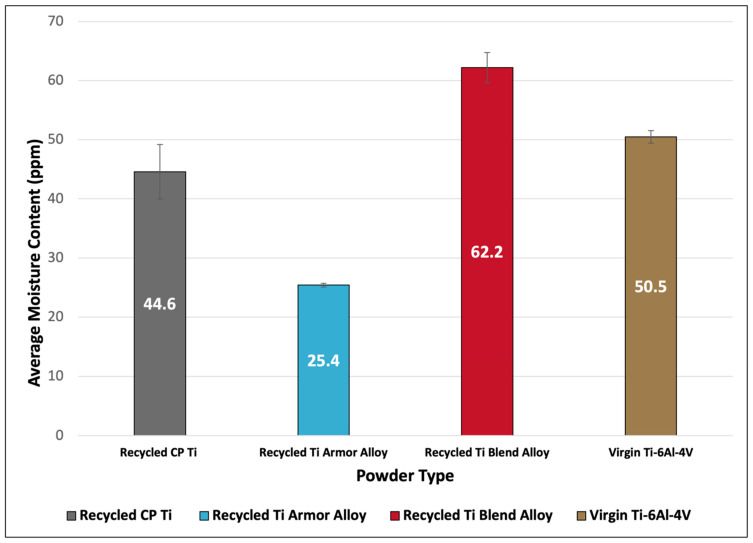
Average moisture content (ppm) of virgin and recycled titanium feedstock powders.

The average moisture content of all titanium powders is displayed in Figure 3. The measured average moisture contents were 44.6 ppm, 25.4 ppm, 62.2 ppm, and 50.5 ppm for recycled CP Ti, recycled Ti amor alloy, recycled Ti blend alloy, and virgin Ti-6Al-4V powders, respectively. While all powders had low moisture contents, both the recycled CP Ti and recycled Ti armor alloy powders interestingly had lower moisture contents than the virgin Ti-6Al-4V. This was unexpected, because it was assumed that similarly to the predictions for O, N, C, and H content, recycled scrap material would have higher moisture content due to increased contaminants or exposure to atmospheric conditions. 

All powders were found to have suitable moisture contents for cold spray applications based on previous laboratory experiences where various material types containing higher moisture contents than those measured in this study were successfully deposited. Additionally, powder exposure studies by Grubbs et al. showed that environmentally exposed Al2024 powder (~320 ppm moisture content) similarly produced highly dense consolidations, <0.2% porosity, when compared to consolidations deposited using dried Al2024 powder (~175 ppm) [20,21]. The combination of these factors validates the usage of both virgin and recycled titanium powders for cold spray. 

It should be noted that all powders (virgin and recycled alike) were stored in a laboratory setting after they were unsealed where there was little impact on the moisture content. Powder moisture content is not a static property and is subject to change if powders are not maintained at proper environmental conditions where the humidity and temperature is controlled. Powder storage plays a substantial role in making sure moisture content doesn’t change due to variations in humidity and weather. These recycled titanium powders have shown to have suitably low moisture contents however, they must continue to be properly stored to ensure there are no substantial increases over time that may further deleteriously increase moisture and subsequently oxide contents. 

### 3.4. Scanning Electron Microscopy (SEM) 

**Figure 4:**
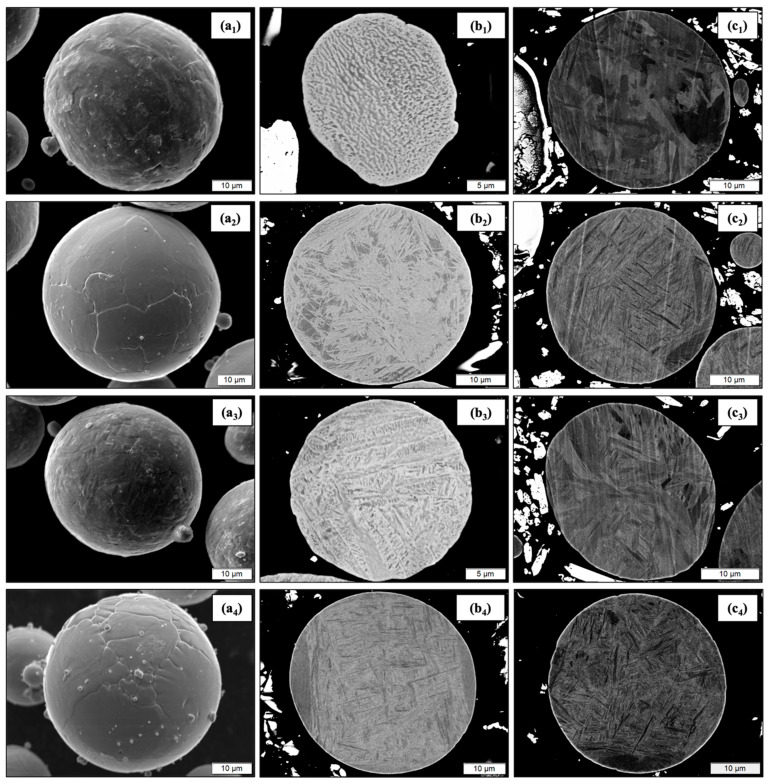
SEM micrographs of loose powder (**a_1–4_**) and polished cross-sections (**b_1–4_**,**c_1–4_**) of Ti CP, Ti armor alloy, Ti blend alloy, and virgin Ti-6Al-4V.

The morphology and microstructure of the titanium powders were further explored through SEM analysis. Figure 4 displays SEM micrographs of loose feedstock powder (a_1_–a_4_), mechanically polished cross-sections of powder particles (b_1_–b_4_), and ion beam polished cross-sections of powder particles (c_1_–c_4_). Superscripts 1, 2, 3, and 4 refer to the recycled CP Ti, recycled Ti armor alloy (Ti-6Al-4V), recycled Ti blend alloy, and virgin Ti-6Al-4V powders, respectively. Figure 4(a_1_–a_4_) loose powder SEM reveals that the plasma and gas atomized titanium powders were predominantly spherical with some smaller satellites on the surface of individual particles. Overall, the SEM reveals similar size, shape, and morphology across powder types, agreeing with PSD analysis. Polished cross-sections of powder particles displayed three unique microstructures with varying degrees of the typical α and β phases found in titanium alloys. The micrographs of recycled CP Ti powder, shown in Figure 4(b_1_,c_1_), highlight the single α-phase present in the microstructure. The α-phase in pure titanium remains stable until the β transus temperature is reached at 883 °C whereby the BCC β phase is formed [22]. The softer α-phase present in the recycled CP Ti powder will promote superior plastic deformation and mechanical interlocking during cold spray which in turn will improve porosity reduction and deposition efficiency. However, improved deposition and denseness of coatings will come at the expense of strength when compared to Ti-6Al-4V or other stronger titanium alloys. As expected, the microstructures of the recycled Ti armor alloy and the virgin Ti-6Al-4V were the same as they share the same alloy chemical composition. There were no visible morphological or microstructural differences between recycled and virgin Ti-6Al-4V powder samples. This was also apparent in the presence of primary α-phases and primary β-phases in Figure 4(b_2_,c_2_,b_4_,c_4_). The β-phases are identified by the lathy, needle-like appearance dispersed within the lighter colored α-phase matrix. This α-β phase combination allows for improved strength and toughness due to the presence β-phases. The increased strength of the recycled Ti armor alloy and virgin Ti-6Al-4V will make it more difficult to achieve dense coatings, requiring a higher gas pressure, temperature configurations, or thermal treatments to achieve the required plastic deformation during the cold spray process [23]. The microstructure of the recycled Ti blend alloy alternatively had a unique appearance because of its origins from both CP Ti and Ti-6Al-4V materials. Microstructural features of the recycled Ti blend alloy were more like that of the recycled CP Ti with fewer features matching that of the Ti-6Al-4V-based powders. It is hypothesized that the recycled Ti blend alloy will act as a “middle ground” between the recycled CP Ti and recycled Ti amor alloy/Ti-6Al-4V when depositing coatings using cold spray. A comparison of the ease of deposition between titanium powder types will be further discussed in Section 3.6 based on the mechanical properties provided by the phases identified. 

### 3.5. X-ray Diffraction (XRD)

**Figure 5:**
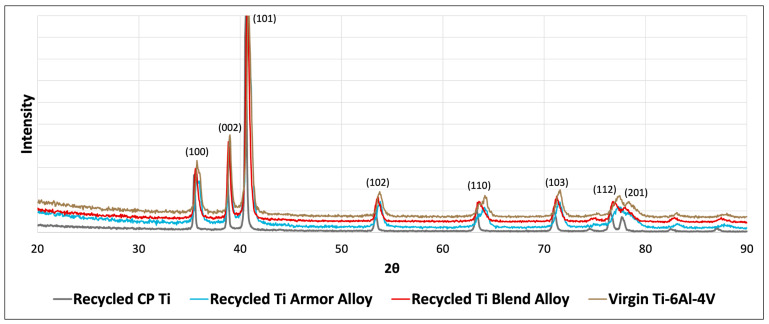
XRD spectra of the four powder samples.

Figure 5 shows the X-ray diffraction spectra results for all four powder samples. These results indicate that there is no difference in secondary phases present within these powders, as all peaks indicate the structure of the titanium matrix. Slight shifting and broadening differences are likely due to differences in chemical composition between the powder samples.

### 3.6. Micro-Particle Compression 

**Figure 6:**
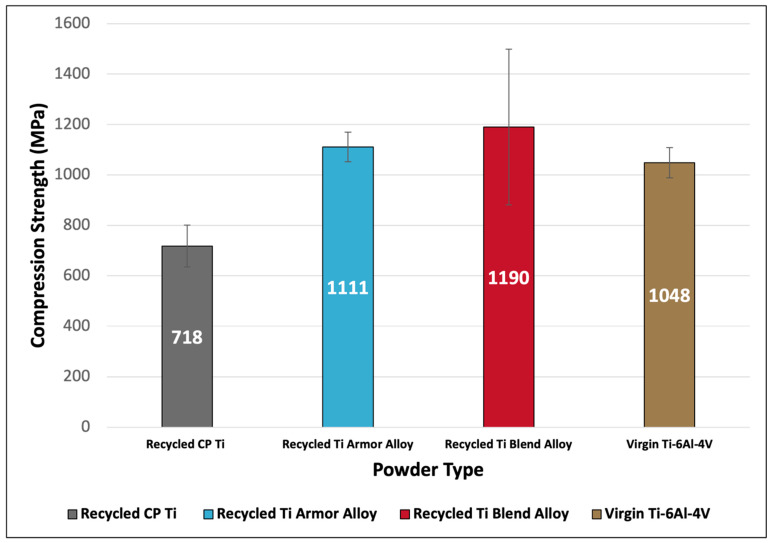
Average particle compression strength of virgin and recycled titanium feedstock powders.

Understanding the compression strength of feedstock powder is essential for predicting how the powder particles will behave during the cold spray process and can greatly improve particle impact simulations [13,14,24]. Figure 6 displays the results of microparticulate compression testing on each of the four titanium powders. The recycled Ti armor alloy, recycled Ti blend alloy, and virgin Ti-6Al-4V powders were all measured to have comparable compression strengths, especially when considering the standard deviations within average measurements. The recycled CP Ti powder had the lowest compression strength (~65% as strong as the other powders) due to a lack of alloying elements that would otherwise mechanically strengthen the powder. The recycled Ti armor alloy compression strength slightly exceeded that of the virgin Ti-6Al-4V powder, highlighting the similarity of mechanical properties between recycled and virgin titanium powders. The Ti blend alloy had the strongest average compression strength by a small margin but had more variation in measurements as reflected by the larger standard deviation. This is thought to be due to the mixed alloyed nature of the Ti blend alloy, leading some particles to be more compositionally and microstructurally analogous to that of the CP Ti or Ti-6Al-4V-based atomization material. The average ultimate tensile strength (UTS) calculated for the recycled CP Ti, recycled Ti armor alloy, recycled Ti blend alloy, and virgin Ti-6Al-4V was 1005 MPa, 1555 MPa, 1666 MPa, 1468 MPa, respectively. Analysis of the calculated powder UTS shows the same trends observed with the average compression strength measurements. Notably, all calculated UTS of titanium powder particles were shown to possess higher UTS values than those of identical wrought or cast titanium alloys [25,26]. This finding is in line with previous reporting that attributes increased powder particle strength to the rapidly solidified nature of atomized powders [13,14,24,27]. The rapid solidification during powder gas-atomization promotes the formation of fine grain sizes that are responsible for increased mechanical strength. 

### 3.7. Nanoindentation 

**Figure 7:**
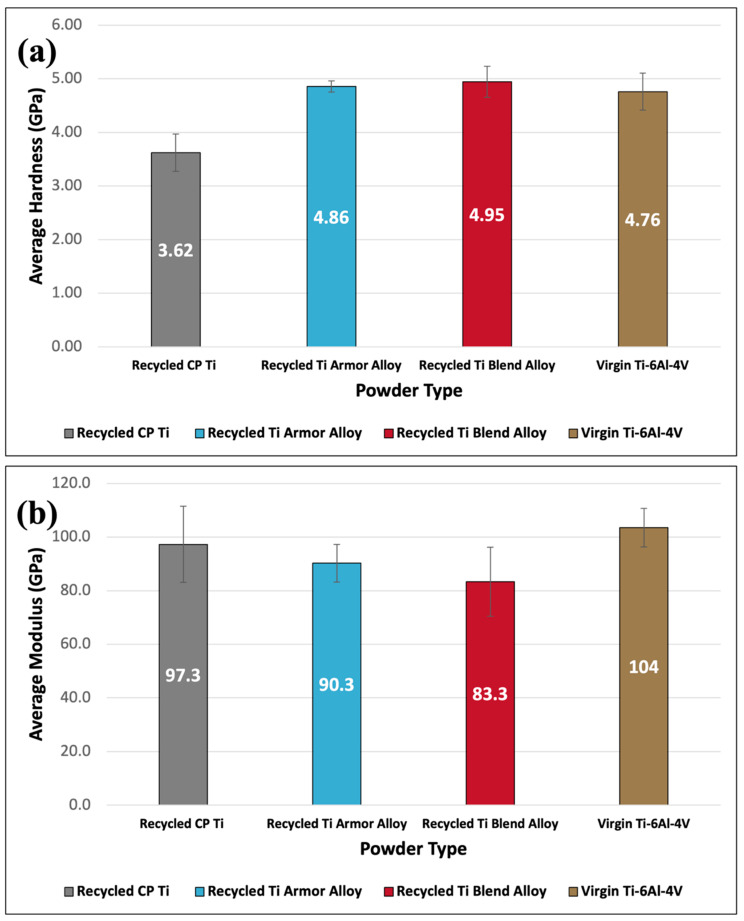
Average dynamic nanoindentation hardness (**a**) and modulus of elasticity (**b**) of virgin and recycled titanium feedstock powders.

Due to the importance of powder microstructure on mechanical properties and, in turn, impact deformation tendencies in cold spray, nanoindentation characterization was used to determine the hardness and modulus of all titanium powders. Figure 7a,b present the average hardness and modulus of elasticity, respectively. As with the average compression strength, the recycled Ti armor alloy, recycled Ti blend alloy, and virgin Ti-6Al-4V powders all had comparable properties with no significant differences in hardness between virgin or recycled powders. The recycled CP Ti powder exhibited the lowest hardness because it lacked hardening elements such as aluminum and vanadium found in Ti-6Al-4V. These elements provide Ti-6Al-4V and similar alloys with solute atoms that distort and interact with dislocations, resulting in improved hardness and bulk strength when compared with pure titanium. Despite containing lower concentrations of titanium and vanadium compared to Ti-6Al-4V, the recycled Ti blend alloy powder exhibited hardness values like those observed for the Ti-6Al-4V based powders. This suggests that the recycled Ti blend alloy has sufficient concentrations of alloying elements to provide comparable strengthening effects to that of Ti-6Al-4V. These results may suggest that factors beyond just titanium and vanadium content, such as distributions of additional alloying solutes, influence the hardness of the recycled Ti blend alloy. 

A contrasting trend was observed between the hardness and modulus values across the recycled titanium powder variants. As shown in Figure 7a, the average hardness increased moving from the recycled CP Ti powder to the recycled Ti armor alloy powder to the recycled Ti blend alloy powder. However, Figure 7b indicates the inverse relationship for average modulus, with the recycled CP Ti powder having the highest average modulus followed by declining values for the recycled titanium armor alloy and recycled titanium alloy blend powders. The inverse relationship observed between hardness and modulus aligns with general materials science principles—namely, the difficulty in simultaneously achieving high strength/hardness and high elasticity in metal alloys. The strengthening mechanisms that increase hardness, such as solute atoms and precipitates obstructing dislocation motion, conversely tend to decrease modulus [28]. However, it is notable that the virgin Ti-6Al-4V powder exhibited the highest overall modulus of elasticity, which does not follow the trend of declining modulus with increasing hardness. This discrepancy suggests additional microstructural or compositional factors may be influencing the modulus versus hardness relationship in the virgin powder. Further investigation is required to understand the mechanisms resulting in the virgin Ti-6Al-4V’s anomalously high modulus. 

## 4. Conclusions

This work characterized the microstructure and mechanical properties of four different titanium powders and explored the feasibility of using recycled titanium feedstock powders for cold spray applications. In this study, recycled CP Ti, recycled Ti armor alloy, and recycled Ti blend alloy powders were all analyzed and compared to virgin Ti-6Al-4V powder using chemical analysis, PSD, SEM, KF Titration, XRD, MCT, and nanoindentation. Analysis of the experimentation and results determined the following conclusions:

- Recycled CP Ti, recycled Ti armor alloy, and virgin Ti-6Al-4V powders all adhered to ASTM-specified composition and impurity standards for their respective alloy compositions. Compliance with the applicable compositional standards verifies the compositional quality of the recycled powder variants and their suitability for use in cold spray processing.
- PSD and SEM results reveal predominantly spherical particles with similar size, shape, and morphology across all powder types. Particle diameters were predominantly ranged between 20–50 μm, ideally sized for cold spray.
- Microstructural features vary between titanium alloys based on slight differences in primary phases identified from XRD.
- Moisture contents of all four titanium powders were found acceptable for cold spray.
- Mechanical testing demonstrated superior hardness and strength in alloyed titanium powders compared to CP titanium. There was no difference in mechanical strength between virgin and recycled powders.
- Recycled titanium powder produced through the atomization of scrap-sourced material provides a viable alternative to virgin titanium alloy powders without compromising mechanical capabilities. Implementing localized or mobile atomization systems to generate recycled titanium powders at a “point of need” enhances operational adaptability and sustainability while potentially decreasing material expenses. This approach provides an avenue to reduce waste, improve cost-effectiveness, and promote circular economy principles for advanced manufacturing.

Building on the findings from this initial study, future efforts will focus on optimizing cold spray parameters to maximize coating density, mechanical strength, and hardness. The four titanium powders analyzed, including the recycled variants, will be deposited using cold spray to assess the viability of applying on-demand recycled feedstock for part repair and replacement applications. Additional exploration into thermal treatments of the powders prior to spraying will also be performed. Specifically, annealing studies on the Ti-6Al-4V powder will be carried out to improve deposition efficiency and minimize porosity by softening the powder before spraying.

## Figures and Tables

**Table 1:** Chemical composition of the four titanium feedstock powders compared with the ASTM F1580-18 standard for CP Ti and Ti-6Al-4V alloys.

Element (wt.%)	O	N	C	H	Al	Fe	V	Cr	Y	Ti
**Recycled CP Ti**	0.162	0.024	0.014	0.0027	0.2	0.093	0.069	0.02	N/A	Balance
**Recycled Ti Armor Alloy**	0.181	0.008	0.004	0.0028	6.04	0.17	4.13	0.014	N/A	Balance
**Recycled Ti Blend Alloy**	0.202	0.015	0.013	0.003	4.35	0.15	2.59	0.018	N/A	Balance
**Virgin Ti-6Al-4V**	0.18	0.01	0.01	0.002	6.35	0.19	3.98	N/A	<0.001	Balance
**ASTM Standard CP Ti** [19]	0.400 (Max)	0.050 (Max)	0.080 (Max)	0.050 (Max)	N/A	0.500 (Max)	N/A	N/A	N/A	Balance
**ASTM Standard Ti-6Al-4V** [19]	0.200 (Max)	0.050 (Max)	0.080 (Max)	0.015 (Max)	5.50–6.75	0.300 (Max)	3.50–4.50	N/A	0.005 (Max)	Balance

## Data Availability

Data will be made available upon request to author.
